# Role of ultrasound evaluation for the diagnosis and monitoring of thyroid tuberculosis: A case report and review of the literature

**DOI:** 10.3892/ol.2014.2652

**Published:** 2014-10-31

**Authors:** GAO-YI YANG, DAN ZHAO, WEN-ZHI ZHANG, JUN MENG, JUN LI, XIAO-HONG LI, HAI-FANG WAN

**Affiliations:** 1 Department of Ultrasonography, Hangzhou Red Cross Hospital, Hangzhou, Zhejiang 310003, P.R. China; 2 Department of Pathology, Hangzhou Red Cross Hospital, Hangzhou, Zhejiang 310003, P.R. China; 3 Department of Anesthesiology, Hangzhou Red Cross Hospital, Hangzhou, Zhejiang 310003, P.R. China

**Keywords:** ultrasound, thyroid tuberculosis, ultrasound-guided core-needle biopsy

## Abstract

Thyroid tuberculosis (TT) is an extremely rare condition, with acute abscess formation being the most uncommon form of presentation. *Mycobacterium tuberculosis* may affect the thyroid gland through hematogenous spread from an extra-thyroid focus of disease or by direct extension from adjacent cervical lymph nodes. Due to the non-specific imaging findings and the variable clinical manifestations, TT is rarely diagnosed promptly prior to percutaneous biopsy or surgery. The present study reports the dynamic monitoring of the sonographic features of a case with thyroid tuberculosis that was diagnosed by a thyroid ultrasound (US) scan, confirmed by a US-guided core-needle biopsy and followed-up sonographically during the whole course of treatment.

## Introduction

Thyroid tuberculosis (TT) is a rare disease even in countries with a high prevalence of other forms of tuberculosis (1). The actual incidence of TT is difficult to assess, but it has been estimated that 0.1–0.4% of all newly diagnosed tuberculosis cases involve the thyroid gland (2–4). Thyroid malignancies, suppurative infections and hemorrhagic cysts are included in the list of the differential diagnoses of TT. Consequently, this infectious disease is often overlooked and patients may suffer from an increased risk of morbidity and mortality due to delayed diagnosis and treatment (5–7). The use of ultrasound (US) occurs extensively in the initial evaluation of thyroid nodules and makes available the relevant information on the characteristics of thyroid lesions and the adjacent parenchyma. Few studies have described the US features of TT to date (8,9). No systematic data have been provided with regard to the evolution of US findings during the course of the disease, from the initial diagnosis until the conclusion of treatment. The present study reports the dynamic monitoring of the US features of a TT case that was diagnosed by US-guided core-needle biopsy (CNB) and followed-up sonographically during the whole course of treatment. Consent was obtained from the patient.

## Case report

A 45-year-old male patient presented to the Hangzhou Red Cross Hospital (Hangzhou, Zhejiang, China) with a painless swelling in the left side of the neck that had gradually increased in size over two weeks. There was no complaint of dysphagia or dyspnea. The patient did not report any history of fever, coughing or hemoptysis, but complained of weight loss of 4 kg within the prior three months. The patient had no past or family history of tuberculosis. Upon examination, a solid, non-tender, 40×30-mm ill-defined mass was present in the left lobe of the thyroid. The cervical lump moved with deglutition, and no enlargement of the regional lymph nodes was observed. The white cell count in the peripheral blood was 10.9×10^9^/l (normal range, 3.0–9.0×10^9^/l), the neutrophil level was 45% and the erythrocyte sedimentation rate (ESR) was 60 mm/h. A thyroid hormonal profile revealed a euthyroid status. Human immunodeficiency virus testing was negative and investigations for infectious or inflammatory conditions were unremarkable. A chest X-ray revealed no pulmonary abnormalities, but the tuberculin test (1:10,000) was strongly positive.

**Figure 1:**
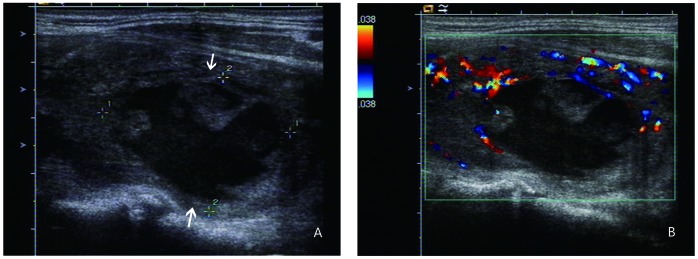
Thyroid tuberculosis in a 45-year-old patient. (A) Sonogram image revealing an ovoid-shaped, heterogeneous, hypoechoic nodule with ill-defined margins in the left thyroid lobe, resembling a cystic nodule with fluid space (white arrow). (B) Color Doppler examination revealing punctated and banded flow signals around the nodule.

US of the thyroid gland was performed using a real-time machine equipped with a 9–11 MHz probe (Philips, Amsterdam, Netherlands). US examination revealed a 34×27×24-mm, ovoid-shaped mass in the left lobe of the thyroid gland. The thyroid lesion was inhomogeneously hypoechoic, with ill-defined margins. An anechoic area with internal hyperechoic spots was present within the mass (Fig. 1A). Color Doppler examination revealed punctate or linear flow signals around the nodule with no intralesional vascularization (Fig. 1B).

**Figure 2:**
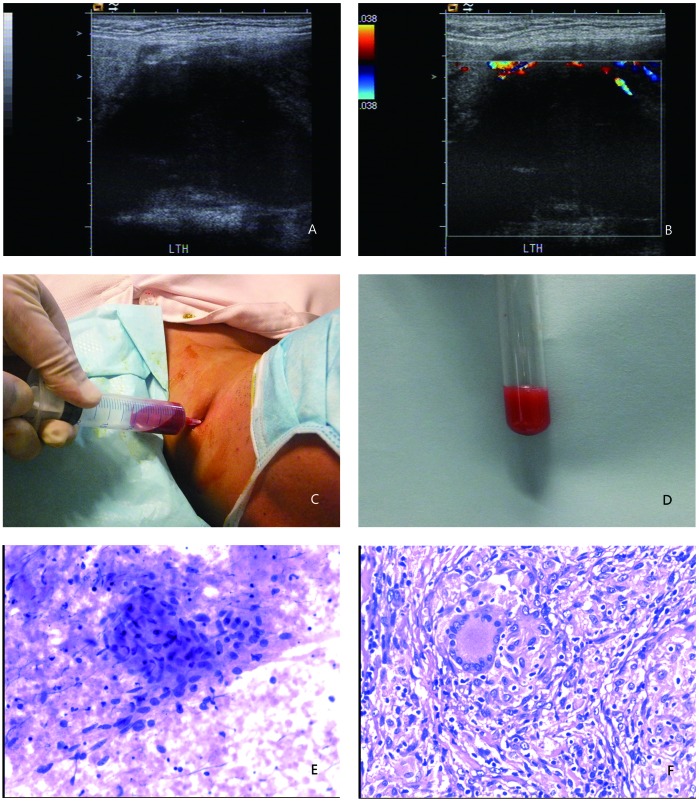
Repeat ultrasound examination at three days post-admission. (A) Longitudinal sonograms revealing a nodule, closely adjacent to the left salivary gland and partially protruding from the upper pole of capsular tissue into the left lobe of the thyroid. (B) A blood flow signal can be observed around the nodule. (C) Fine-needle aspiration cytology of the nodule. (D) The aspiration yielded reddish pus that was revealed to be red blood cells with necrosed materials. (E) Follicular epithelial cells on a bloody background, with clusters of purple-stained colloid and a field of red-stained granular caseous necrosis. (F) Multifocal granulomatous nodules inbetween atrophic thyroid follicules, with fibrous tissue proliferation and chronic inflammatory cell infiltration. (E anf F: stain, hematoxylin and eosin; magnification, ×200).

The patient’s general condition worsened three days after admission. There had been an increase in the size of the swelling, associated with a 37–38°C low-grade fever. A repeat US examination demonstrated that the mass in the left lobe of the gland had increased in volume to 65×35×38 mm. The lesion partially protruded from the upper pole of the left thyroid lobe (Fig. 2A). Compression with the probe and positional changes demonstrated the presence of liquid within the mass. Blood flow signals were observed by color Doppler examination around, but not inside the lesion (Fig. 2B). A US-guided fine-needle aspiration (FNA) biopsy of the thyroid mass yielded a thick, caseous and bloody material (Fig. 2C and D). Microscopic examination revealed the presence of follicular epithelial cells in flaked or honeycombed formations on a bloody background, together with clusters of purple-stained colloid and a field of granular caseous necrosis (Fig. 2E). In order to obtain a histological diagnosis, a US-guided CNB of the adjacent solid tissue was performed with a 21-gauge cutting needle. Histopathological examination revealed that multifocal granulomatous nodules were distributed in between atrophic thyroid follicles, chronic inflammatory cell infiltration and fibrous tissue proliferation (Fig. 2F). Acid-fast staining on frozen-sections did not indicate the presence of bacteria. However, the presence of multifocal granulomatous inflammation associated with caseous necrosis was strongly suggestive of tuberculous infection.

**Figure 3:**
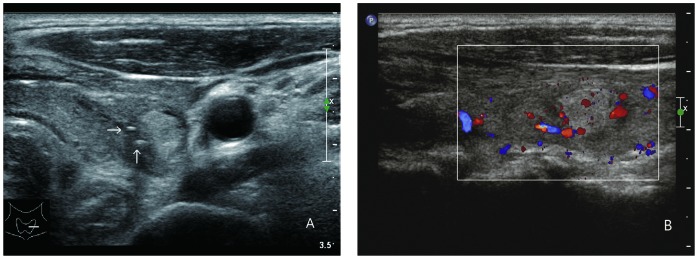
Repeat ultrasound examination six months after the initiation of anti-tuberculosis medication. (A) Significant shrinkage and echo enhancements can be observed. The nodule is solid and well-defined, with two tiny calcifications inside (white arrows). (B) Color Doppler examination revealing punctate and strip-shaped blood flow around and inside the nodule.

The patient was administered an oral three-drug regimen of antituberculous medication (0.3 g isoniazid, 0.6 g rifampicine and 0.2 g ciprofloxacine, once daily for a total of 3 months). Subsequent to three months of treatment, US demonstrated clinically significant shrinkage of the nodule down to 16×10×12 mm and the appearance of coarse calcifications inside the lesion. A repeat US examination was performed six months later without any marked changes in size, but with progressive loss of the definition of the nodule limits (Fig. 3). The patient remains without clinical symptoms at the time of writing.

## Discussion

Tuberculosis affects almost all organs of the human body, but involvement of the thyroid gland, although initially reported in 1863, is rare (1). Resistance to tuberculous infection due to the bactericidal action of thyroid colloid, the high blood flow and tissue oxygenation, and the high concentration of iodine within the gland are possible explanations (10).

The clinical presentation of TT is non-specific and variable (11,12). TT may be asymptomatic or may present with an occasionally elusive spectrum of manifestations. Physical examination may reveal an isolated nodule, as observed in the present case, a nodular goiter or a cold abscess. Laboratory examination may also fail to provide a substantial clue for the exact diagnosis. In the present study, laboratory examination only noted a slight increase in the white blood cell count and ESR, which were not specific for TT. Consequently, the diagnosis of TT is frequently delayed and may represent an incidental finding at pathological examination. By contrast, an unnecessary thyroidectomy may occur in cases mimicking a fast-growing thyroid malignancy (4,13).

US-guided FNA biopsy of the lesion appears to be a useful tool for the timely and accurate diagnosis of TT (14,15). In the present case, however, the initial FNA biopsy revealed only non-diagnostic necrotic material. US-guided CNB of the intra- and perilesional solid component made the correct diagnosis of TT possible. Notably, the microbiological evaluation of FNA specimens for *Mycobacterium tuberculosis* was negative in the present case. This finding highlights the pivotal role of US-guided histopathological examination for a timely diagnosis.

A variable spectrum of US findings from few TT cases have been sporadically reported in previous studies. Kang *et al* (9) presented a case in which US examination revealed multifocal, heterogeneous, hypoechoic lesions with ill-defined margins in each lobe of the thyroid. Kang *et al* (8) performed US examinations on two cases, one of which demonstrated an enlarged right lobe of the gland and a well-defined lesion that was predominantly anechoic, with internal echoes and irregular margins. In the other case, a large, heterogeneous, predominantly anechoic lesion with an irregular wall and few internal echoes in the left lobe was revealed. All these studies highlight the importance of using US in arriving at an accurate diagnosis of TT. In the present case, the initial US examination revealed a heterogeneous, fluid-filled nodule coexisting with internal bleeding, which was hardly distinguishable from a colliquated thyroid adenoma. The repeat US examination revealed progressive changes in the US findings until the nodule was solid and hyperechoic with ill-defined margins. Two, tiny, calcified foci were also found inside the mass, which made this lesion similar to calcified tubercular goiters. These changes in the US examination may be of use in monitoring the efficacy of antitubercular treatment and suggest the importance of dynamic US monitoring for TT patients.

TT should be considered in the differential diagnosis of neck masses, particularly in those lesions that increase rapidly in size and resemble thyroid malignancy. US examination and US-guided CNB are important techniques for a timely and accurate diagnosis of thyroid malignancies. These rapid, safe and inexpensive diagnostic procedures can prevent an unnecessary thyroidectomy and make an appropriate antitubercular therapy possible.
